# Differential Mitochondrial Genome Expression of Three Sympatric Lizards in Response to Low-Temperature Stress

**DOI:** 10.3390/ani14081158

**Published:** 2024-04-11

**Authors:** Jingyi He, Lemei Zhan, Siqi Meng, Zhen Wang, Lulu Gao, Wenjing Wang, Kenneth B. Storey, Yongpu Zhang, Danna Yu

**Affiliations:** 1 College of Life Science, Zhejiang Normal University, Jinhua 321004, China; hejingyi58@zjnu.edu.cn (J.H.); 2022205000889@zjnu.edu.cn (L.Z.); mengsiqi@zjnu.edu.cn (S.M.); wangzhen@zjnu.edu.cn (Z.W.); gaolulu@zjnu.edu.cn (L.G.); wwj1660@zjnu.edu.cn (W.W.); 2 Department of Biology, Carleton University, Ottawa, ON K1S 5B6, Canada; 3 College of Life and Environmental Science, Wenzhou University, Wenzhou 325035, China; 4 Key Lab of Wildlife Biotechnology, Conservation and Utilization of Zhejiang Province, Zhejiang Normal University, Jinhua 321004, China

**Keywords:** low-temperature stress, lizard, *RT*-qPCR, mitochondrial genome expression

## Abstract

**Simple Summary:**

Global climate change is a growing threat to the lives and ranges of reptile species. The present study examines three lizard species (*Calotes versicolor*, *Ateuchosaurus chinensis*, and *Hemidactylus bowringii*) that share a sympatric distribution in the Guangzhou region of Guangdong, China. Mitochondria act as energy-producing centers, and the present study analyses the effects of low temperatures in the context of warm winters on lizards, by examining their responses to low-temperature stress at the level of mitochondrial gene expression. This present study analyses the effects of low temperatures in the context of warm winters on the mitochondrial gene expression of lizards and reveals the implications of the distribution within it.

**Abstract:**

Ecological factors related to climate extremes have a significant influence on the adaptability of organisms, especially for ectotherms such as reptiles that are sensitive to temperature change. Climate extremes can seriously affect the survival and internal physiology of lizards, sometimes even resulting in the loss of local populations or even complete extinction. Indeed, studies have shown that the expression levels of the nuclear genes and mitochondrial genomes of reptiles change under low-temperature stress. At present, the temperature adaptability of reptiles has rarely been studied at the mitochondrial genome level. In the present study, the mitochondrial genomes of three species of lizards, *Calotes versicolor*, *Ateuchosaurus chinensis*, and *Hemidactylus bowringii*, which live in regions of sympatry, were sequenced. We used *RT*-qPCR to explore the level of mitochondrial gene expression under low-temperature stress, as compared to a control temperature. Among the 13 protein-coding genes (PCGs), the steady-state transcript levels of *ND4L*, *ND1*, *ATP6*, and *COII* were reduced to levels of 0.61 ± 0.06, 0.50 ± 0.08, 0.44 ± 0.16, and 0.41 ± 0.09 in *C. versicolor*, respectively, compared with controls. The transcript levels of the *ND3* and *ND6* genes fell to levels of just 0.72 ± 0.05 and 0.67 ± 0.05 in *H. bowringii,* compared with controls. However, the transcript levels of *ND3*, *ND5*, *ND6*, *ATP6*, *ATP8*, *Cytb,* and *COIII* genes increased to 1.97 ± 0.15, 2.94 ± 0.43, 1.66 ± 0.07, 1.59 ± 0.17, 1.46 ± 0.04, 1.70 ± 0.16, and 1.83 ± 0.07 in *A. chinensis*. Therefore, the differences in mitochondrial gene expression may be internally related to the adaptative strategy of the three species under low-temperature stress, indicating that low-temperature environments have a greater impact on *A. chinensis,* with a small distribution area. In extreme environments, the regulatory trend of mitochondrial gene expression in reptiles is associated with their ability to adapt to extreme climates, which means differential mitochondrial genome expression can be used to explore the response of different lizards in the same region to low temperatures. Our experiment aims to provide one new research method to evaluate the potential extinction of reptile species in warm winter climates.

## 1. Introduction

In recent years, average global temperatures have generally risen, particularly winter temperatures that are creating a warmer climate. However, extreme cold days within a warm winter have also occurred more frequently on the Asian continent. Climate cooling in winter would be more detrimental to the survival of amphibians and reptiles than climate warming in winter [1]. Against a background of a warm winter climate, if extreme cold weather episodes persist in the future, this will become the major factor affecting the structure of subtropical ecological communities. Due to climate warming, the boundary of species distribution moves northward, and the influence of extreme cold weather on subtropical communities and ecosystems can become more pronounced, and will exert a strong natural selection on wild populations [2,3]. With the increasing frequency of extreme weather, non-seasonal, short-term, and severe temperature changes may seriously interfere with the survival and population stability of animals, especially leading to a decline of reptile species diversity [4,5]. Climate change may enhance ectothermic metabolism and embryonic development, which can reduce survival and life expectancy, thereby increasing vulnerability to climate change [6] and affecting the fitness of reptile species at different latitudes for various reasons [7,8]. The existence and advancement of human society depend heavily on species diversity, which is also crucial to preserving ecological equilibrium. At present, research on the ecological effects of extreme low-temperature events is insufficient, and the impact of warm winter climate change is often neglected [3]. Hence, it is necessary to explore the response of reptiles to extreme weather from multiple perspectives and provide corresponding scientific strategies for better species protection.

Reptiles are ectotherms. When they enter hibernation, their hearts, brains, livers, and skeletal muscles adopt a respiratory protection strategy of reverse thermal compensation, which reduces metabolic rates, enhances the antioxidant activity of tissues, maximizes energy saving, and reduces heat production requirements with minimal food supply [9,10]. In particular, changes in gene expression levels have attracted much attention. For instance, under extremely cold conditions, the gene expression levels of carboxylic ester hydrolase and sodium symporter in the liver of *Anolis carolinensis* are significantly different, which is an important reason for the loss of physiological function and individual deaths of *A. carolinensis* caused by extreme low temperatures [11]. The transcription factor C/EBPA increased, whereas numerous transcriptional regulators, including KDM6B and JARID2, were downregulated when the temperature dropped [12]. The expression of the cold-induced mRNA binding protein (CIRBP) and HSPA8 mRNA in the lizard liver decreased significantly under cold stress [13]. Hence, the importance of gene expression differences in reptile adaptation to low-temperature stress has been increasingly confirmed.

Sympatric species in different distribution areas can show different responses to the same environmental stress, to separate niches, reduce interspecific competition, and ensure survival [14]. For example, in a comparison of adaptive responses to temperature by three lizard species (*Eremias argus*, *Eremias multiocellata*, and *Phrynocephalus przewalskii*) that are distributed in the same region, it was found that there were different temperatures and microhabitat preferences, as well as preferred body temperatures, under different distribution backgrounds [15]. As the sensitivity of reptiles to temperature changes, they can be used as an ideal model for studying the cold-tolerance mechanisms of different species in regions of sympatry.

Mitochondria are the main sites of oxidative phosphorylation and ATP formation, and are responsible for energy conversion, coordinating cell metabolism, development, and aging, and participating in numerous life activities, including responses to hypothermia [16]. Mitochondrial genes also have more applications in studying the phylogenetic relationships of species, particularly where gene rearrangements are significant [17]. The mitochondrial structures of the three lizards (*Calotes versicolor*, *Ateuchosaurus chinensis*, and *Hemidactylus bowringii*) used in this experiment have been characterized and used to resolve their taxonomic status [18,19,20]. However, the lack of research on mitochondrial genomic expression changes is often overlooked. To date, studies of the effects of temperature change on reptile growth and gene expression levels have focused mainly on physiological responses and differences in nuclear gene expression, but few have examined changes in mitochondrial genome expression. The mitochondrial genome harbors the genetic code for 13 proteins and forms the core constituents of mitochondrial respiratory complexes I–IV that are imbedded in the inner mitochondrial membrane [21]. Wood frogs, *Rana sylvatica*, are capable of enduring total-body freezing throughout the winter season. One feature of freezing survival proved to be a strong upregulation of transcripts of the mitochondrial genes *ATP6/8*, *ND4*, and *16S RNA* during wood frog freezing (24 h at −2.5 °C) in the liver and brain [22]. However, the relative transcript level of the *COI* gene decreased in a study of the response of *Dryophytes versicolor* mitochondrial PCG to freezing stress [23]. In contrast to nuclear genes such as HSPs, which are involved in sensing temperature stress, no such representative genes have been proposed in the mitochondrial genes of lizards. Mechanisms for coping with heat or cold stress vary among species. Comprehensive findings may be obtained by conducting experiments using the whole mitochondrial genome. In a study of killifish, Healy et al. analyzed the expression of the mitochondrial genome to identify responsive genes, using RNA-seq [24]. A similar attempt was made in frogs to study cold stress [25].

**Figure 1:**
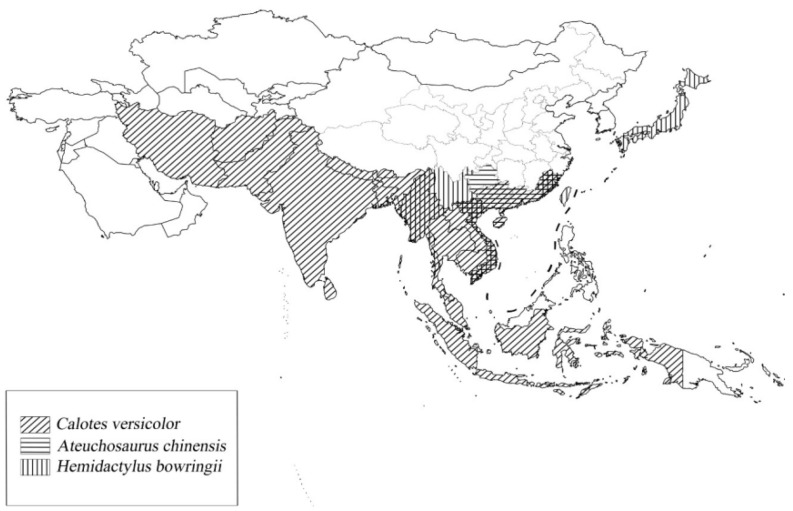
Main distribution areas of *Calotes versicolor*, *Ateuchosaurus chinensis*, and *Hemidactylus bowringii* in southeastern China, southwestern Asia, and southeastern Asia. *A. chinensis* is found mainly in southern China and Vietnam; *H. bowringii* is found mainly in southern China, Vietnam, Burma, and Japan; and *C. versicolor* is found widely across Asia.

In the present study, we analyzed changes in the expression of mitochondrial genes in three common Asiatic lizards. *Calotes versicolor* (oriental garden lizard) is distributed mainly in southeastern China, southwestern Asia, and southeastern Asia. *Ateuchosaurus chinensis* (Chinese short-limbed skink) is found in southern China and Vietnam. *Hemidactylus bowringii* (Oriental leaf-toed gecko) occurs mainly in southern China, Vietnam, and Burma (Figure 1). These three species are distributed together in Guangzhou, Guangdong Province, China. Their cold tolerance mechanisms may have both differences and commonalities, and so we aimed to investigate whether the responses of these species to climate change could differ among taxonomic, spatial, or environmental properties. Currently, according to a primitive perspective, the ability of many species to endure global changes in environmental temperatures is linked to their present level of plasticity for fitness-related traits [26,27,28]. Therefore, the climatic variability hypothesis (CVH), as a mainstream view, suggests that, since the range of climatic fluctuations that terrestrial animals experience throughout a year rises with latitude, individuals at higher latitudes should have a greater range of thermotolerance plasticity, thus allowing them to respond more readily to fluctuating environmental conditions, i.e., more seasonal changes in the environment [28,29]. From this, we speculate that there may be a commonality of regulatory mechanisms that contribute to cold tolerance in terrestrial ectothermic animals. Factors that affect the level of climate change in a habitat can be factors that affect the cold tolerance of species. Thus, we hypothesized the following: (1) reptile species living at low latitudes and low altitudes are more susceptible to low-temperature stress, and (2) reptiles with a small distribution range in subtropical regions are more susceptible to low-temperature stress. To simulate sudden low temperatures in a warm winter climate, we set 25 °C as a normal temperature simulation of a warm winter climate, whereas a temperature of 4 °C or 8 °C was used to simulate low temperatures. This study explores the adaptive mechanisms seen in reptile mitochondrial genomes in different distribution areas and the possible internal relationships to low-temperature stress. What is more, we explore the impact of low temperatures on species in the context of a warming winter climate and also consider the impact of mitochondrial genome expression differences on species diversity.

## 2. Materials and Methods

### 2.1. Sample Collection, Acclimatization, and Low-Temperature Stress

Groups of 40 lizards for each species (*C. versicolor*, *A. chinensis*, and *H. bowringii*) were acquired from Guangzhou, Guangdong Province, China. We made sure that the lizards were basically the same in terms of form and size, sex, age, health, etc. [30] in order to guarantee that the samples were as similar as possible. All the lizards were acclimated at 25 °C in 100 cm × 80 cm × 100 cm terrariums for one week, while being fed on mealworms, crickets, and cockroaches to ensure a stable survival rate. Lizards were then divided into ambient control groups (25 °C) and low-temperature groups (4 °C), with 10 animals in each group. All samples in the ambient control group were held at 25 °C for 24 h, whereas the low-temperature groups of *C. versicolor*, *A. chinensis*, and *H. bowringii* were held at 4 °C for 24 h [31]. However, the low-temperature groups (4 °C) of *A. chinensis* and *H. bowringii* had a large number of deaths under a 4 °C exposure, with *A. chinensis* showing a total death result. To further clarify the mechanisms of cold tolerance in *A. chinensis* and *H. bowringii*, we set up a second low-temperature group (at 8 °C), with 10 samples of the two lizard species held at 8 °C for 24 h.

### 2.2. DNA Extraction, PCR Amplification, and Sequencing

Although the mitochondrial genome of the three species involved in this study has been previously characterized, we sequenced the genomic DNA of the sampled populations to obtain the whole genomes. This was done to ensure the accuracy of the *RT*-qPCR data in subsequent experiments and to eliminate any interference caused by inter-population differences. Whole genomic DNA was extracted from the 5 mm tip of the tail of the three species, lysed by proteinase K, and purified using the Ezup Column Animal Genomic DNA Purification Kit (Sangon Biotech Company, Shanghai, China) [32], to obtain the full mitochondrial genome sequence. Then the extracted DNA was separated using 1% agarose gel electrophoresis [33]. Using common primers for lizards designed by Kumazawa [34], we modified eleven pairs of primers (Table 1) to amplify multiple sub-segments and designed specific primers to complete the remaining gaps using Primer Premier 5.0 [35]. The mitochondrial genes of the three species of lizards were obtained via PCR amplification and sequence assembly. The fragment amplified by primer “ND2-CO1L; ND2-CO1H” was used for sequencing *COX1* [36], ensuring that the haplotype differences in the mitochondrial genes were under 0.01 [37]. For positioning tRNA genes, we used the MITOS service (http://mitos.bioinf.uni-leipzig.de/index.py, accessed on 14 Nov 2023) [38]. Using Mega 7.0, combined with the mitochondrial genome published on the NCBI (Accession No. KM508815, AB183287, MW327509) and the acquired lizard mitochondrial genome, the 13 PCGs, 2 rRNAs, and the control region [39] were used for manual positioning and annotated in SnapGene Viewer http://www.snapgene.com/, accessed on 14 November 2023 [40].

### 2.3. mRNA Extraction and cDNA Synthesis

In the experimentally treated groups of *C. versicolor*, *A. chinensis*, and *H. bowringii*, 10 specimens from six groups, including the ambient control groups (25 °C) and cold-acclimated groups (4 °C or 8 °C), were placed on a pre-cooled autopsy plate and dissected. Sample livers were rapidly dissected, their condition recorded, frozen in liquid nitrogen and held in a −80 °C freezer [13,41,42,43,44]. RNA from the liver of four samples for each of the six groups was extracted and purified using an Animal Tissues Total RNA Extraction Kit (Forgene Company, Chengdu, Sichuan, China). Then a PrimeScript™ RT Reagent Kit with gDNA Eraser and a PrimeScript™ RT Master Mix kit (Takara, Dalian, Chin were used to remove the genomic DNA from the extracted RNA sample, followed by reverse transcription of RNA into DNA [45], ensuring that only cDNA was detected as a template for accurate gene expression analyses. The operation of this reaction was conducted on ice to ensure the accuracy of the system.

### 2.4. RT-qPCR Primer Design and Reaction

Fluorescent quantitative primers were designed for the 13 PCGs using Primer Premier 5.0 [35], according to the complete sequences of *C. versicolor*, *A. chinensis*, and *H. bowringii* that were obtained from the routine PCR. The sequence of the *β-actin* gene [44] served as the internal reference gene. The primers shown in Table 2 were screened by *RT*-qPCR reactions. The samples in the cold and ambient groups that were ultimately used for quantitative analyses were confirmed based on the analysis of the genetic divergence of the *COI* gene. Referring to the qPCR reaction performed by Cai et al. and Wang et al. [25,46], the reaction procedure was the following: 95 °C for 30 s, followed by 40 cycles of 55 °C for 5 s and 95 °C for 30 s, with a final hold at 4 °C. Due to the qPCR reaction being quite demanding in terms of spiking, all systems except the cDNA template were first prepared in EP tubes, shaken, mixed well, and configured in an amount slightly larger than that required for the experiment to ensure a sufficient reaction solution. 

### 2.5. qPCR Data Analyses

A stranded qPCR approach was employed to determine transcript levels for the 13 mitochondrial PCGs in the samples. For the purpose of statistical analyses, StepOne Software v2.2.2 (Applied Biosystems, Foster City, CA, USA) and the “Comparative C_T_ (ΔΔ C_T_)” program were used. The transcript level was reflected by the RQ value, with each sample repeated three times. An independent sample *t*-test was performed to analyze differences between RQ values using Microsoft Excel, in which a significant difference was expressed as *p* < 0.05 and a highly significant difference as *p* < 0.01 [47]. The determination of low temperature-sensitive genes was based on a numerical analysis of changes in mitochondrial gene expression before and after the low-temperature treatment of conspecific lizards. The degree of significance of gene expression regulation was analyzed by comparing the *p* values. Finally, Origin 8.0 software [48] was used for data analyses and chart making.

## 3. Results

### 3.1. Quantitative Analyses of Mitochondrial PCGs

**Figure 2:**
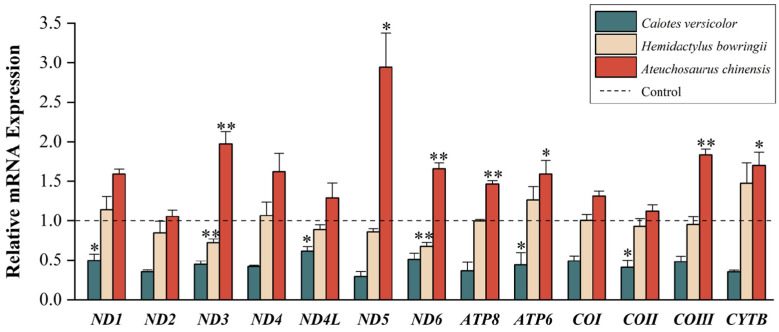
The steady-state transcript levels of 13 PCGs in the three lizard species in response to low-temperature stress. Gene names are displayed on the *x*-axis and gene steady-state transcript levels are shown on the *y*-axis. The dashed line shows controls, standardized to 1.0, dark green columns show the experimental group for *C. versicolor* (at 4 °C), light yellow columns show the experimental group for *H. bowringii* (at 8 °C), and red columns show the experimental group for *A. chinensis* (at 8 °C), all after 24 h exposure to low temperatures. Asterisks indicate significantly different expressions as compared with controls (*, *p* < 0.05) and (**, *p* < 0.01).

After 24 h of exposure to low temperatures (4 °C for *C. versicolor*, 8 °C for *H. bowringii* and *A. chinensis*), the motility of the three lizard species had declined significantly, which indicated that acute cold treatment had produced a pronounced physiological response in the lizards. For the lizards treated for 24 h at room temperature (25 °C) or at low temperature (4 °C for *C. versicolor*, 8 °C for *H. bowringii* and *A. chinensis*), *RT*-qPCR was adopted to identify the steady-state transcript levels of the 13 PCGs in the liver mitogenome (Figure 2).

The experimental results for *C. versicolor* treated to low-temperature exposure showed that transcript levels of the 13 PCGs declined in response to cold exposure. Using a *t*-test, the transcript levels of the *ND4L, ND1, ATP6*, and *COII* genes showed statistically significant reductions (*p* < 0.05) to values of 0.61 ± 0.06, 0.50 ± 0.08, 0.44 ± 0.16, and 0.41 ± 0.09, respectively, as compared with controls. The experimental results for *H. bowringii* treated to low temperatures showed fewer effects of cold exposure on transcript levels, with significant cold-induced reductions for the *ND6* and *ND3* genes (*p* < 0.01) to levels of 0.67 ± 0.05 and 0.72 ± 0.05, respectively, as compared with controls. Contrary to the results for the above two species, the data for *A. chinensis* exposed to low temperatures showed a significant upregulation of transcript levels for *ND5*, *Cytb*, and *ATP6* (*p* < 0.05), with values of 2.94 ± 0.43, 1.70 ± 0.16, and 1.59 ± 0.17 over controls, respectively. The remaining four PCGs (*ND3*, *ND6*, *ATP8*, and *COIII*) also showed statistically significant increases (*p* < 0.01) in transcript levels, contributing to increased values of 1.97 ± 0.15, 1.66 ± 0.07, 1.46 ± 0.04 and 1.83 ± 0.07, respectively, over controls and showing that those genes may be the important genes to defend against low temperatures.

### 3.2. Comparison of Cold Tolerance Plasticity

As a direct result of low-temperature stress in the previous experiments, *C. versicolor* showed slow movement but could still tolerate the sudden drop in temperature and the continuous 4 °C low temperature. However, this was not the case for the other two species of lizards. After 24 h of exposure to 4 °C, the *H. bowringii* group showed a large number of individual deaths, whereas all *A. chinensis* individuals died. This indicated that 4 °C of low-temperature stress significantly affected the mobility of *C. versicolor* and severely interfered with the normal physiological and biochemical processes of *H. bowringii* and *A. chinensis*, leading to death. However, both *H. bowringii* and *A. chinensis* survived at 8 °C but showed slow movement and stiffness of the trunk, which indicates that they were able to tolerate the temperature drop from 25 °C to 8 °C and continuous stress at 8 °C for a reasonable amount of time. Based on the above results, it is clear that 4 °C is a critical temperature for *H. bowringii*, but a lethal temperature for *A. chinensis*. Hence, it can be inferred that *C. versicolor* had the strongest cold tolerance plasticity, whereas *A. chinensis* showed the worst cold tolerance plasticity among the three lizards.

## 4. Discussion

### 4.1. Low-Temperature Stress on Mitochondrial Gene Expression

Mitochondria, as semi-autonomous organelles found in the cytoplasm of eukaryotic organisms, play an important role in oxidative phosphorylation and ATP synthesis and are also closely involved in biological signal transmission, cellular differentiation, metabolism, cell growth, senescence, and apoptosis. However, in comparison to nuclear genes, mitochondrial genes show a higher sensitivity to environmental coercion [49,50].

By studying the relative mitochondrial gene expression of the lizard *C. versicolor*, it was found that this species exhibited lower metabolic activity as compared to the other two lizard species under low-temperature conditions. Mitochondrial oxidative phosphorylation-related genes were significantly downregulated in expression, including the *ND4L*, *ND1*, *ATP6*, and *COII* genes, all of which are important genes encoding respiratory chain enzyme complexes [51]. *ND1* and *ND4* are key components of the highly hydrophobic subunits within the membrane arm of complex I. These subunits are essential for the early assembly of this complex and are also involved in the regulation of complex I, including control over the rate of protein degradation [52]. *COII* encodes the second subunit of cytochrome c oxidase (complex IV), which is one of the three subunits responsible for the core functional formation of complex IV [53]. The subunit encoded by the *ATP6* gene is the key ingredient of the proton channel and the subunit of ATP synthase that plays a direct role in the trans-membrane transport of protons [54]. A decrease in the expression levels of these genes directly affects the activity of respiratory chain-related enzymes such as cytochrome c oxidase, blocking electron transportation in mitochondria, and reducing electron transfer activity, redox-driven activity, and transmembrane transporter protein activity to varying degrees [55]. A strategy that reduces metabolic activity controlled by mitochondria moderates oxidative phosphorylation [56] and redox reactions in the respiratory chain, minimizing energy consumption in order to survive in cold conditions.

Several mitochondrial PCGs were downregulated under low-temperature stress in *H. bowringii,* and downregulation of the expression of the *ND3* and *ND6* genes was particularly pronounced. The proteins encoded by these two genes are essential components of complex I within the mitochondrial respiratory chain, enabling NADH dehydrogenase activity that participates in mitochondrial electron transport by transferring electrons from the NADH to ubiquinone. The downregulated expression of the *ND3* and *ND6* genes results in lower activity of mitochondrial complex I and may be associated with a defective assembly of the entire respiratory complex, generating more proton leakage and fewer oxidative phosphorylation couplings. This can lead to a reduced membrane potential difference, resulting in less ATP production and less deleterious reactive oxygen species, which may help some species to combat cold environments [57]. It has been shown that poikilothermic animals can suppress their metabolic rate, thereby reducing their need for endogenous fuel reserves and prolonging their survival in harsh environments, including cold climates [58]. This is similar to the adaptive mechanism of Antarctic fish that live in a persistently cold climate [59]. The above studies suggest that temperature may enhance organismal adaptation by reducing the expression of key genes in the respiratory chain complex and thus decreasing respiration rates.

Exposed to low temperatures, the expression levels of multiple mitochondrial PCGs in *A. chinensis* showed a significant upward trend. At low temperatures, reptiles can resist cold damage by acclimatory adjustments in metabolic efficiency [60]; for instance, the organism can regulate the activity of the complex in the respiratory chain. *A. chinensis* may resist cold damage by increasing the expression of mitochondrial genes to acquire an intense stress state, instead of decreasing the expression of the mitochondrial genes and entering into a state of hibernation. Cold acclimation increased oxidative capacity, increased mitochondrial content, and enhanced the activity of functional enzymes in the oxidative respiratory chain; however, the mitochondrial decoupling pathway showed no changes [61]. In addition, the expression level of the *COIII* gene of *A. chinensis* is significantly elevated under low-temperature treatment. Related studies have shown that a reduced temperature limits membrane fluidity, but mitochondria are able to maintain membrane function by changing the percentage of unsaturated fatty acids in the mitochondrial membrane. That may result in changes in the activities of COX enzymes, succinate dehydrogenase, and NADH dehydrogenase, potentially causing an upregulation of crucial genes within the respiratory chain complex [62]. Membrane remodeling contributes to changes in mitochondrial oxidative activity and promotes the activity of membrane-associated metabolic enzymes. The associated molecular mechanisms may be similar to a known process where the activity of COX in the muscle of carp rapidly increases during cold acclimation [63].

Referring to the work of Wang [46] on low-temperature gradient stress at 2 °C and 4 °C, in which transcript levels of some mitochondrial genes of a frog (*Fejervarya kawamurai*) from Guangzhou, China, were significantly downregulated at 4 °C, it was suggested that these frogs entered a state of hypometabolic dormancy. Given that PCG expression levels showed a tendency to increase in response to low temperatures at 2 °C, we hypothesized that a similar response existed in the lizards. After low-temperature stress, mitochondrial gene expression was reduced in *C. versicolor* and *H. bowringii* and increased in *A. chinensis.* This indicated that temperature stress had the greatest effect on *A. chinensis*.

In the study of adaptation to temperature stress in lizards, the heat shock proteins (HSPs) family has received a lot of attention. Upregulation of HSP gene expression under heat stress has been confirmed in numerous studies, and its response to temperature increase is stable [64,65,66]. However, under cold stress, there are contradictory results, showing a downregulation of HSPs gene expression [13] as well as results of an unchanged expression [67]. The connection between its expression and the response of lizards to low temperatures remains unclear. The mechanism of lizard adaptation to low temperature is complex, one in which a variety of genes such as CIRBP (Cold-Inducible RNA Binding Protein), ROR (RAR-related orphan receptor gamma), and PER (Period Circadian Regulator) are functioning [13]. Changes in mitochondrial gene expression in lizards under cold stress may be a new clue to studying the mechanism of cold adaptation.

### 4.2. Differential Gene Expression of Different Lizards in Regions of Sympatry

Different lizard species in regions of sympatry may have different adaptive mechanisms to the same environmental factors, such as differences in their thermal preferences. Compared to high thermal preferences, low thermal preference usually has a lower critical minimum and occurs in species from colder regions and/or colder seasons [68]. Cold tolerance in reptiles has been shown to correlate with their latitudinal and altitudinal ranges, and cold environments at high latitudes or altitudes can promote greater cold tolerance among regional poikilothermic vertebrates [8,69]. This is consistent with our speculation based on the climatic variability hypothesis. Within regions of high latitude, animals show greater thermal tolerance plasticity because of a more variable seasonal climate. Therefore, reptiles distributed in low latitudes and altitudes could be more susceptible to acute cold stress as a result of their poorer cold tolerance plasticity. Of the species analyzed in the present study, *H. bowringii* is distributed at the highest latitude, whereas *A. chinensis* is found at lower latitudes and *C. versicolor* has the lowest latitude distribution, living in a warm and humid environment. If the differences in cold tolerance plasticity are determined by the latitude of the distribution area, then the potential experimental results should be that *H. bowringii* has the strongest cold tolerance plasticity and *C. versicolor* the weakest. This is inconsistent with our findings that all *A. chinensis* specimens were killed during pre-laboratory treatment at 4 °C, whereas the other two species did not show mass death. Thus, the differences in cold tolerance plasticity of these lizards were not decided solely by the latitude of the distribution area.

The key to the climatic variability hypothesis is that strong seasonal temperature differences in habitats enhance the temperature tolerance plasticity of species. However, seasonal temperature variations are not only due to differences in latitude but are also determined by the size of a species range. The larger the distribution range, the higher the habitat complexity experienced by a species, and this may require a stronger adaptive capacity to acute change in environmental temperature in diverse habitats. Johannes et al. demonstrated that species from a wider range have a higher temperature tolerance plasticity than species from a single hot and humid tropical climate, even under the same temperatures [70]. Among reptiles, Cowles similarly noted that at low temperatures, lizards with narrow distribution ranges are less adapted to their environment than other lizard species with wider territorial ranges [71]. In the present study, the distribution ranges of *C. versicolor* were from 10° S to 38° N, 44 to 140° E, whereas *A. chinensis* ranged from 13 to 29° N, 100 to 109° E, and *H. bowringii* was found from 13 to 46° N, 92 to 145° E [72,73,74,75]. The different distribution areas had different environmental conditions, and the variations in ecological factors may lead to some diverse responses to low-temperature environmental coercion among these species [76], which is mainly focused on metabolic adaptation. After seasonal low-temperature environmental acclimation in the various regions, the local populations showed different degrees of cold tolerance plasticity. *C. versicolor* had the largest distribution area among the three species, occurring over a wider range of both latitude and longitude. Its populations were acclimated under complex low-temperature environmental stress conditions in different microhabitats within the distribution area, resulting in populations that were more responsive to temperature. Populations could even adapt to low-temperature stress by lowering their metabolic activity until they entered a dormant state. Species with larger distribution ranges would also encounter more habitat extremes and, therefore, the ranges of widespread populations could lead to a greater tolerance of variable environmental conditions [77]. Due to its small distribution range with a warm and humid climate and a small seasonal temperature change, the population of *A. chinensis* is poorly fitted to deal with substantial changes in temperature outside of its normal range and is unable or limited in the use of dormancy to withstand low temperatures. The distribution range of *H. bowringii* is wider than that of *A. chinensis,* and *H. bowringii* is better acclimated to a cool climate in the mid-latitude region, so it displays a stronger cold tolerance than *A. chinensis*. However, compared with *C. versicolor*, the habitat of *H. bowringii* is more homogeneous, and its adaptive ability to the environment is weaker. Its response mechanism forms an intermediate type, in which the upregulation and downregulation of the expression of various genes coexisted, but downregulation is the most significant, which may decrease ATP and reactive oxygen species production and lower metabolic rates to fight against the cold environment. The three sympatric lizards differed significantly in gene expression under low-temperature stress, differed in cold-tolerance mechanisms, and mostly used a mixed strategy [78] to survive the winter. This suggests that the three species differed in cold tolerance plasticity and that sympatric species in the context of different distributions may be able to effectively mitigate or avoid interspecific competition in the context of limited resources via thermal–ecological segregation. Different environments pose different selection pressures and provide opportunities for behavioral and physiological adaptations to different temperature extremes [79]. Typically, species with long, widely varying environmental ranges show higher plasticity or tolerance [80]. In summary, *C. versicolor* has the strongest cold tolerance plasticity and *A. chinensis* has the weakest plasticity when exposed to low temperatures under winter conditions. This is consistent with the fact that all *A. chinensis* died under our pre-laboratory 4 °C low-temperature treatment, whereas the other two species did not show a substantial mortality. Under low temperatures in the context of a warm winter climate, it is assumed that the reptile species most affected are those with an increased mitochondrial gene expression under low-temperature stress. By contrast, reptile species with smaller distribution ranges and monoculture habitats are less cold-tolerant and more susceptible to the effects of low-temperature stress.

All in all, we can draw a preliminary result about the factors influencing the cold tolerance of lizards. The second hypothesis is supported by our experimental results: reptiles with a small distribution range in a subtropical region are more susceptible to low-temperature stress. Fundamentally, it is the complexity of the habitat that influences the cold tolerance of lizards, with larger ranges generally representing a wider variety of climates and microhabitats that can spawn greater cold tolerance plasticity.

Given the impact of climate on the distribution of lizards, it is expected that lizards would shift their range northward due to global warming. However, the fact is that there are eastward, westward, and northward shifts in the center of the distribution, and shifts in the boundaries of the distribution in all four directions [81]. Lizards from different distributions show different patterns of adaptation to climate change, which are correlated with the thermal quality of the distributions [82]. It is clear that the influence of the distribution area on the species makes them show various abilities to adapt to climate change. The thermal tolerance of similar species from different distributional areas differs [83], which confirms that the acclimatization to distributional environments can affect the plasticity of species. Studies on the thermal adaptation in lizards have also concluded that widely distributed species are more plastic than narrowly distributed species [84,85]. The vulnerability shown by micro-endemic species in response to climate change has been mentioned in several articles [86,87,88]. During the process of species adaptation to climate change, the mechanisms are specific, such as altering organ size and mitochondrial, proteomic, and metabolomic regulation [84]. However, the link between the size of a species’ distribution area and its ability to adapt to climate change is universal.

### 4.3. Conservation Strategy for Lizards

Most conservation strategies for lizards are based on geographically distinctive species or isolated populations [89,90,91], which are characterized by small or fragmented habitats. Based on this study, this characteristic is one of the most important reasons for their endangerment: lizards with small ranges are unable to cope with climate change. Taking together the conservation strategies that have been proposed for lizards [89,91,92], we propose the following conservation strategies to address this feature: (1) Determine the current habitat distribution of the study species and assess how the habitat is changing—is it shrinking, degrading or fragmenting? (2) Conserve habitat. Because of similarities in general patterns of habitat selection among lizard species [93], habitat shrinkage can be restored to some extent based on lizard habitat preferences. In a smaller distribution area, under long-term single microclimate domestication, lizards may go to a more dangerous situation, and climate variability will be fatal to them. (3) Population replenishment and habitat expansion. Population densities are uneven within habitats, and, generally speaking, population densities are lower in the peripheral areas of habitats and are affected by the migration of individuals, which will further lead to habitat shrinkage. Population replenishment—i.e., the transfer of individuals from high-density areas to low-density areas or areas of reduced habitat—may be able to control this decline, gradually restoring habitat areas and reducing the loss of genetic variability, which also reduces the likelihood of extinction of the entire population. (4) Predict the risk of species extinction. Characteristics of the distribution area can be an important predictor of species extinction risk.

## 5. Conclusions

Above all, the experimental results have indicated that the association between cold tolerance plasticity and latitude is not absolute. Because of the different sizes of distribution areas and the multiplex habitat microclimates experienced, there may be different mechanisms for the low-temperature protection of these lizards from different geographical populations. In addition, their abilities to endure low-temperature stress varied. *C. versicolor,* with the largest distribution range, had the strongest cold tolerance plasticity, whereas *A. chinensis,* with the smallest distribution range, showed the worst cold tolerance plasticity. Under extremely low temperatures against a warm winter background, *A. chinensis* was unable to adapt well to rapidly changing temperatures and showed a greater likelihood of extinction. At the biomolecular level, after being subjected to low-temperature stress, the expression levels of multiple genes encoding subunits of the 13 proteins in the mitochondrial genome of *C. versicolor* and *H. bowringii* showed significant downward trends, and *A. chinensis* showed significant upward trends, compared to the control group. The above results support the second hypothesis that reptiles with a small distribution range in subtropical regions are more susceptible to low-temperature stress. Accordingly, the mitochondrial genes of lizards are more sensitive to environmental low-temperature stress factors, and their expression trends can be developed as a monitoring tool to determine whether species are prone to extinction. Therefore, studying the trend of mitochondrial genome expression changes under low-temperature stress can provide a comprehensive synthesis of data support for exploring tolerance plasticity and broaden our thinking on reptile diversity protection. However, the response mechanism of lizards to low-temperature environments in different habitats still needs further exploration.

## Figures and Tables

**Table 1:** Modified universal primer details for sequencing the mitochondrial genomes in this study [34]. Note: W = A/T, R = A/G, K = G/T, Y = C/T, S = G/C, M = A/C, B = C/G/T, D = A/G/T, H = A/C/T, N = A/C/G/T.

No.	Primer Name	Nucleotide Sequence (5′ to 3′)	Amplicon Size (bp)
1	xiyi-12S-J505	ACAAACTAGGATTAGATACCC	730
	xiyi-12S-N1225	CANBTTTCCCTTGCGGTACT	
2	16SL	AACCCYYGTACCTYTTGCATCATG	886
	16SH	TCCACAGGGTCTTYTCGTC	
3	ND1-ND2L	CGATTTCGCTATGACCAACT	891
	ND1-ND2H	ATTGATGAGWAKGCTATRATTTTTCG	
4	ND2-CO1L	GCCCCMYTMCACTTCTGA	1142
	ND2-CO1H	GTAHAGGGTGCCRATRTCTTT	
5	SX-ND5-ND6-J	GARCARGACCTYCGACTAATRGG	1067
	SX-ND5-ND6-N	ATATTAGTAGTGTTTGTSTATAC	
6	CO1-CO2L	TACTCAGACTACCCAGAYGC	971
	CO1-CO2H	CCRCARATTTCTGAGCATTG	
7	ND4-CUNL	CCMAAAGCCCAYGTAGAAGC	909
	ND4-CUNH	CTTHTACTTGGADTTGCACC	
8	SX-ND5-GLU-J	YTYATTAACGCCTGAGCCTT	805
	SX-ND5-GLU-N	ATAACAACGAYGGTTTTTC	
9	CYTB-ProL	TGAGGACAAATATCMTTCTGAGG	861
	CYTB-ProH	TTAAAATKCTAGTTTTGG	
10	Thr-CRL	YAAAGCMTTGRTCTTGTAA	1635
	Thr-CRH	CTCGAKTTTWGGGGTTTGRCGA	
11	CR-12SL	TCGYCAAACCCCWAAAMCGAG	627
	CR-12SH	TRTAACCGCGGTKGCTGGCAC	

**Table 2:** Primer sequences of 13 mitochondrial PCGs and *β*-actin gene designed for the *RT*-qPCR experiment described in this study. Note: “DLLX” means *C. versicolor*, “DLGX” means *A. chinensis*, and “DLXH” means *H. bowringii*.

Primer Name	Forward Primers (5′ to 3′)	Reverse Primers (5′ to 3′)	Amplicon Size (bp)
DLLX-*β*-ACTIN	GCTCTGCTATGTTGCCCTTG	ACCTGAACCGCTCATTACCA	125
DLLX-ND1	TCCTTCTTAGTAGCCGTAGCA	TCCGTCTGCCATTGGTTGA	123
DLLX-ND2	CCTCATGCCTGCTTCTCCTA	GGTTAGTAGTGTGCTGCCTTG	182
DLLX-ND3	GCCCTACGAATGCGGATTT	ACAGTTGGTGTTGGTGCTAG	156
DLLX-ND4	CTAACCAACCTGGCACTTCC	GGTGGCTGAGGCAATAATTGT	105
DLLX-ND4L	CTAACACTAAATACCCCCCAC	AGAAGAGGTGACGAGGAGTGT	102
DLLX-ND5	GCCACAGCAGGAAGTCTTCT	GCTTGGAGTGCGGATGAGT	119
DLLX-ND6	CGGTGGCGTGTTATTATTCG	ACCAGCACCAACAATTAGGAG	173
DLLX-ATP6	CGCCTGACCGCTAACCTAA	TGGATGAGTGCTACGGCTATT	155
DLLX-ATP8	GCAACTTAACCCAAACCCAT	TTGGATTTGTGGTTGTGGTG	104
DLLX-COI	CTTGTGAGCCTTCTTGTACGA	TTCCGAAGCCGCCGATTA	145
DLLX-COII	CACGACTACGCCATAACTACC	TCGGTTAGTACGGTGGTGAA	101
DLLX-COIII	GCCAATTCTAGCCGCCATATC	TGTGCCTTCACGAATGATGTC	151
DLLX-CYTB	TAGCCGCCTCAGTCCTAATC	CCGCTTGGTTGTCCTCCTA	151
DLGX-*β*-ACTIN	AAGGAGAAGCTGTGCTATGTG	AGGAAGGAAGGCTGGAAGAG	164
DLGX-ND1	GACCATCCTCCTCTTCACCAA	AGTAGACCGCAGTGCTTGATA	159
DLGX-ND2	CCGAGCAACAGAAGCAACAA	AAGTATCGTACAGGCGTAGGG	145
DLGX-ND3	AACCCTCCCAGACACAGAAA	GCAAGAATAGGATGGCGACTA	113
DLGX-ND4	CCTTCTCCGCCACAGACTT	TAGCCGTTCAGCTTGATTACC	104
DLGX-ND4L	GCTGCCTACCAACACAATGT	GTTCTGTATGTGGTCGGTTCC	121
DLGX-ND5	CAAGACCGCCTTATCACTCTG	TGCTAGTTGTGGTTGGTTGAG	166
DLGX-ND6	GGACCCGTATCCTGAGACT	GCACGAATCAACCCAAATC	162
DLGX-ATP6	TACCAGAAGGCACTCCTACAC	GCTGTGAGGTTGGCAGTTAG	112
DLGX-ATP8	ATGCCACAACTAAATCCCG	TTGATTTGGGTTGAGGCTG	104
DLGX-COI	GCTCCACGACACTTACTACG	GCCTGCGAATATTACTCCGAA	163
DLGX-COII	CAGACTACGAGGACCTGTTGT	ACGGCTCACGAGTGGAGAA	163
DLGX-COIII	GGCTTCGCTACGGAATAGTC	GTTAATGCCGCTTGGAGGTC	134
DLGX-CYTB	CCTTGTCATAGCCACAGCATT	CGCCTCAGATCCACTCTACTA	137
DLXH-*β*-ACTIN	GAGGGAGATTGTGCGGGATAT	AGGAAGGACGGCTGGAAGA	186
DLXH-ND1	TCGCCGTAGCATTCCTGAC	GTTGTTGGTCGTGTTGGTTCT	151
DLXH-ND2	CACCATACCACCAGCACTAAT	TAAGGCAACCAGGAGTCACC	142
DLXH-ND3	TCCCATTCTCAATACGCTTCTT	TGTTGTAAGGTGTAGTGTTGTG	131
DLXH-ND4	AGCCTGTATAGCCGCACTAC	GATGATTCCGTATCCGCCAAG	142
DLXH-ND4L	AACTATAAGCACCACCACAGC	TTAGGTTGTCCGAGGCGTAT	125
DLXH-ND5	ATCCGCACCTACCACGATTC	CGGTTGAAGATTACGGCTTGAA	196
DLXH-ND6	GGGATGCTTGTTGTGTTTGC	AACCACCGCCTCCATTACA	175
DLXH-ATP6	ATCGCACAACAGCACTACAG	GGTGAAGGTATATGGCAGGAG	161
DLXH-ATP8	TATAACTGCTGTCGTCACCTG	CTGATTGTGTTGATGGGTTTGG	101
DLXH-COI	CTCGCCGCTACTCTGACTAC	GCTGAGAAGTGTGGTTGATGTT	158
DLXH-COII	CCGCCTCACCAACCATAGA	GGGCAGCACAGTTCAAATAGT	173
DLXH-COIII	GCAAGCGATAGAGTACGGAGA	CCAGGCATACAGTGAGGAATG	131
DLXH-CYTB	ACCACCACATATTAAGCCAGAG	GCGACTGATATAAGGAGTGCTA	105

## Data Availability

The data supporting the findings of this study are openly available from the National Center for Biotechnology Information at https://www.ncbi.nlm.nih.gov (accessed on 20 December 2023). The accession numbers are PP003926, PP003925, and OR991118.
